# Antiatherogenic and Cardioprotective Effects of Black Chokeberry (*Aronia melanocarpa*) Juice in Aging Rats

**DOI:** 10.1155/2015/717439

**Published:** 2015-08-13

**Authors:** Elena Daskalova, Slavi Delchev, Yulia Peeva, Lyudmila Vladimirova-Kitova, Maria Kratchanova, Christo Kratchanov, Petko Denev

**Affiliations:** ^1^Department of Anatomy, Histology and Embryology, Medical University of Plovdiv, 15A Vassil Aprilov Boulevard, 4002 Plovdiv, Bulgaria; ^2^Department of Social Medicine and Public Health, Medical University of Plovdiv, 15A Vassil Aprilov Boulevard, 4002 Plovdiv, Bulgaria; ^3^Medical University of Plovdiv, Clinic of Cardiology, 66 Peshtersko Shosse Boulevard, 4002 Plovdiv, Bulgaria; ^4^Institute of Organic Chemistry with Centre of Phytochemistry, Bulgarian Academy of Sciences, Laboratory of Biologically Active Substances, 139 Ruski Boulevard, 4000 Plovdiv, Bulgaria; ^5^Innovative-Technological Centre (ITC) Ltd., 20 Dr. G. M. Dimitrov Street, 4000 Plovdiv, Bulgaria

## Abstract

Age-related diseases are a social problem of global significance and their prevention by natural products is a research area of particular interest. The present study is an approach to counteract the risk factors for atherosclerosis arising in the aging process by supplementation of chokeberry juice. It employed a model of healthy adult rats monitored for a number of somatometric, serum lipidogram, and histopathological parameters, related to risk factors and their response to supplementation with antioxidant-rich chokeberry juice. The results were used to calculate different atherogenic and cardioprotective indices, and all results were compared to those of young healthy rats. Chokeberry juice proved an extremely rich source of polyphenols resulting in very high antioxidant activity. Treatment with *Aronia* juice significantly lowered the proatherogenic low-density lipoprotein fraction of the animals studied and led to a 16.5% decrease in their total cholesterol. Atherogenic indices in *Aronia*-supplemented animals clearly showed lower atherogenic risk and cardioprotective indices indicated protection of the cardiovascular system. Besides that, chokeberry juice retarded the age-related changes in the aortic wall and can be recommended as a prophylactic tool for healthy aging.

## 1. Introduction

The aging of world population has been considered as a relatively new process for the human history. Long life span and declining fertility rates are among the causes responsible for the greater number of people over 60 in most countries [1]. Aging is defined as a time-dependent decline of functional capacity and stress resistance, associated with increased morbidity and mortality. Diabetes, obesity and overweight, high blood pressure, neurodegenerative diseases, and mainly cardiovascular complications are considered serious health problems in the elderly. The free radical theory of aging hypothesizes that oxygen-derived free radicals are responsible for the age-related damage at cellular and tissue levels. In a normal situation, a balanced equilibrium exists among oxidants, antioxidants, and biomolecules. Excess generation of free radicals may overload the natural cellular antioxidant defenses, leading to oxidation and further contributing to cellular functional impairment. The identification of free radical reactions as promoters of the aging process implies that interventions aimed at limiting or inhibiting them should be able to reduce the rate of age-related changes with a consequent reduction of aging rate and disease pathogenesis [2]. Practically, all tissues can undergo the biochemical and cellular alterations of aging and this process is accompanied by changes in the morphology and physiology of different organs. Cardiac damage represents one of the main health problems associated with aging and obesity-induced morbidity and mortality, but the triggering mechanisms are not completely clear [1]. One of the preeminent theories of heart aging involves cellular damage associated with cumulative damage from reactive oxygen species (ROS) and reactive nitrogen species (RNS). The high metabolic activity of the heart is supported by a large population of mitochondria and a steady supply of oxygen. Moreover, cardiomyocytes are replaced infrequently, making them ideal candidates for oxidative damage and stress with the passage of time [3]. Age-related structural alterations in the walls of large arteries, including the aorta, cause a decrease in the total arterial compliance, which in turn leads to both a decreased distal blood flow and an increased aortic pulse pressure. The latter has been shown to be the strongest predictor of cardiovascular mortality, because it increases the mechanical load on the left ventricle [4]. Nutrition has been recognized to have an important impact on overall mortality and morbidity and its role in extending life expectancy has been an object of extensive scientific research [5]. A growing amount of evidence indicates that the consumption of plant foods is correlated with a lower risk for development of arteriosclerosis and oxidative stress-related diseases [6]. In contrast, diets poor in plant-based foods and rich in animal products are related to an increased risk for cardiovascular diseases [7]. Most of the antioxidants taken with the diet are of plant origin and the richest sources are herbs, cereals, fruits, and vegetables in which polyphenol substances, carotenoids, vitamin C, and vitamin E are the biggest contributors to their antioxidant activity. Recently, the physiological effects of polyphenol-rich foods have received a lot of attention as dietary sources of antioxidants that are valuable for human health. Many epidemiological studies have strongly suggested that there is a correlation between intake of polyphenol-rich foods and low mortality due to coronary heart disease (CHD). Myocardial infarction and ischemic stroke, closely related to atherosclerosis, are major causes of death in advanced countries [8]. Taking into account the association of oxidative stress and various pathological conditions, much attention is being paid to dietary antioxidants as alternative therapeutic agents [9]. In search of novel sources of dietary antioxidants, black chokeberry (*Aronia melanocarpa*) is very appropriate because it is one of the richest sources of polyphenols among fruits. It belongs to the Rosaceae family and is cultivated as an ornamental shrub and used as raw material for juices, wines, jams, and so forth, as well as a source of natural food colorants. The high polyphenol content of* Aronia* fruit places it among the sources with highest* in vitro* antioxidant activities [10]. There is an appreciable experimental evidence of* Aronia* effectiveness in a broad range of pathological conditions mediated by uncontrolled oxidative processes, which reveals a broad range of health effects including antimutagenic, anticancer, antidiabetic, antihypertensive, hepatoprotective, and immunomodulating effects [10, 11]. The mechanisms of the* in vivo* antioxidant activity of* Aronia* polyphenols after absorption spreads out far beyond radical scavenging and includes suppressing the formation of ROS and RNS, inhibition of prooxidant, and restoration of antioxidant enzymes, and probably also cellular signaling, to regulate the level of antioxidant compounds and enzymes [10]. Although the lipid lowering properties of chokeberry have already been reported [12–16], its effects on the processes of aging have not been addressed. The present study presents an approach to counteract the risk factors for atherosclerosis arising in the aging process by supplementation of chokeberry juice. It employed a model of healthy adult rats monitored for a number of somatometric, serum lipidogram, and histopathological parameters, related to risk factors and their response to supplementation with antioxidant-rich chokeberry juice. The results were used for the calculation of different atherogenic and cardioprotective indices. Besides, histological examination of thoracic aorta of tested animals was performed and all results were compared to those of young healthy rats.

## 2. Material and Methods

### 2.1. Black Chokeberry (*Aronia melanocarpa*) Fruit Juice

Commercially available sterilized black chokeberry juice, packed in glass bottles (250 ml), was provided by Vitanea Ltd., Plovdiv, Bulgaria. According to the food regulations, the term “fruit juice” is used for unfermented product obtained from healthy, ripe, fresh, chilled, or frozen fruit (one or more kinds) with the typical fruit color, flavor, and taste. Therefore, all carbohydrates and antioxidants investigated in the current study present naturally in the black chokeberry fruit. The chemical composition and antioxidant activity of* Aronia* juice are presented in Tables 1 and 2.

### 2.2. Animals

The study included 18 male Wistar rats; 12 of them aged 10 months with initial body weight 350 g ± 50 g; 6 animals aged 2 months with body weight 100 g ± 10 g. Animals were bred in the vivarium of the Medical University, Plovdiv, under standard laboratory conditions (housed in polypropylene cages in an environmentally controlled clean-air room with a temperature of 22 ± 3°C, a 12-h light/dark cycle, and a relative humidity of 60 ± 5%). Taking into account the hormonal influences in female, we used only male rats. The rats were divided into 3 groups including 2 control groups: (1) six 2-month-old rats defined as control young (CY) and six 10-month-old defined as control old (CO), which were on a standard diet and tap water* ad libitum*. The animals of the third experimental group (A) received* ad libitum* chokeberry juice diluted 1 : 1 in drinking water and a standard rodent chow (containing 13.45% protein, 51.6% carbohydrate, 3.40% fat, and 2908 kcal/kg of metabolizable energy). The daily dose of fruit juice ingested by the animals was 25 ml, corresponding to 64 ml/kg. The experiment lasted 90 days [17].

Rats were kept according to all experimental procedures recommended by the European Commission for the protection and welfare of laboratory animals. The experimental protocol was approved by the Committee on Ethical Treatment of Animals of the Bulgarian Agency for Food Safety. In the end of experiment, after measurement of body weight (g) animals were euthanized with i.p. application of T61. The abdominal circumference (cm) and body length (cm) were measured before decapitation. Total blood was collected by a funnel into a centrifuge tube and allowed to clot to obtain serum. The thoracic aortas were separated for histological examination.

### 2.3. Anthropometrical Determinations

The body weight and body length were used to determine the following anthropometrical parameters:

- Body mass index (BMI): body weight (g)/length^2^ (cm^2^)
- Lee index: cube root of body weight (g)/nose-to-anus length (cm) [18].

### 2.4. Histology

Descending thoracic aortas from CY, CO, and A groups of rats were harvested and fixed in 10% formalin for 48 hours and then transferred to 70% ethanol for storage at 4°C. Fixed aortic segments were embedded in paraffin and sectioned at 5 *μ*m thickness. Sequential sections were stained with hematoxylin/eosin and orcein. The photomicrographs enclosed were taken on Nikon Microphot SA microscope (Japan), equipped with a Camedia-5050Z digital camera (Olympus, Japan).

### 2.5. Biochemical Determinations

Blood was placed in a centrifuge tube and allowed to clot to obtain the serum. Serum was separated by centrifugation at 1400 g for 10 min. The triacylglycerol (TG, mmol/l) and total cholesterol (TC, mmol/l) were assayed in serum by enzyme-colorimetric method; high-density lipoprotein cholesterol (HDL-C, mmol/l) and low-density lipoprotein cholesterol (LDL-C, mmol/l) were assayed in serum by direct enzyme-colorimetric method (Beckman Coulter chemistry analyzer AU 480). The atherogenic indices were determined as TC-C/HDL-C ratio, LDL-C/HDL-C ratio. The cardioprotective indices were determined as HDL-C/LDL-C and HDL-C/TC-C ratio [19–23]. Non-HDL-C was calculated as TC-C − HDL-C [24] and remnant cholesterol (remnant C) was calculated as TC − HDL-C − LDL-C [25].

The percent reduction or increase of serum lipid fractions was calculated as

(1) $$\begin{array}{c} \text{\% reduction or increase}=\frac{\left(A-B\right)}{B}\times 100, \end{array}$$

where *A* = serum values of controls and *B* = serum values of treated group [26].

### 2.6. Characterization of Black Chokeberry Juice

#### 2.6.1. HPLC Analysis of Phenolic Compounds

High Performance Liquid Chromatography (HPLC) analyses of phenolic components were performed on Agilent 1220 HPLC system (Agilent Technology, USA), equipped with binary pump and UV-Vis detector. Wavelength of *λ* = 280 nm was used. Phenolics separation was performed using Agilent TC-C18 column (5 *μ*m, 4.6 mm × 250 mm) at 25°C. Mobile phases constituted 0.5% acetic acid (A) and 100% acetonitrile (B) at a flow rate of 0.8 ml/min. The gradient condition started with 14% B (0–6 min), linearly increased to 25% B (6–30 min) and then to 50% B (30–40 min). The standard compounds (gallic acid, 3,4-dihydroxybenzoic acid, chlorogenic acid, caffeic acid, p-coumaric acid, ferulic acid, ellagic acid, catechin, epicatechin, rutin, naringin, myrecetin, quercetin, naringenin, and kaempferol) were purchased from Sigma-Aldrich (Steinheim, Germany). Neochlorogenic acid was calculated as equivalents chlorogenic acid.

#### 2.6.2. HPLC Analysis of Sugars

HPLC determination of sugars was performed on Waters 484 system, connected to a refractometric Waters R401 detector and Aminex HPX–87H column (300 × 7.8 mm, BioRad), eluent 0.004 mol/l H_2_SO_4_, flow 0.5 ml/min, and temperature 23°C. The standard compounds (glucose, fructose, sucrose, and sorbitol) were purchased from Sigma-Aldrich (Steinheim, Germany).

#### 2.6.3. Total Polyphenol Compound Analysis

Total polyphenols were determined according to the method of Singleton and Rossi (1965) with Folin-Ciocalteu's reagent [27]. Gallic acid was employed as calibration standard and results were expressed as gallic acid equivalents (GAE) per liter juice.

#### 2.6.4. Total Anthocyanins Determination

Anthocyanins were determined by the pH-differential method [28]. The absorption of the sample was measured at pH 1.0 and pH 4.5 and the difference in absorbance was proportional to the anthocyanin content. The total anthocyanin content (TAC) was expressed as mg cyanidin-3-glucoside equivalents per liter juice and calculated via the following formula:

(2) $$\begin{array}{c} \text{TAC}\left(\;\text{mg}/\text{l}\;\right)=\frac{A\times \text{MW}\times \text{DF}\times 1000}{\varepsilon \times L}, \end{array}$$

where *A* = (*A*_510 nm_ pH 1.0−*A*_700 nm_ pH 1.0)−(*A*_510 nm_ pH 4.5−*A*_700 nm_ pH 4.5); MW = cyanidin-3-glucoside molecular weight (449.2); DF = dilution factor; *ε* = cyanidin-3-glucoside molar absorptivity (26900); *L* = cell pathlength (usually 1 cm).

#### 2.6.5. Total Proanthocyanidin Content Analysis

Total proanthocyanidin content was determined by the method of Sarneckis et al. (2006) [29]. The results were expressed as milligrams of catechin equivalents (CE) per liter of juice.

#### 2.6.6. Determination of Antioxidant Activity by Oxygen Radical Absorbance Capacity (ORAC) Assay

ORAC was measured according to the method of Ou et al. (2001) [30] with some modifications described in detail by Denev et al. (2010) [31]. The method measures the antioxidant scavenging activity against peroxyl radical generated by thermal decomposition of 2,2′-azobis[2-methyl-propionamidine] dihydrochloride (AAPH) at 37°C. Fluorescein (FL) was used as the fluorescent probe. The loss of fluorescence of FL was an indication of the extent of damage from its reaction with the peroxyl radical. The protective effect of an antioxidant was measured by assessing the area under the fluorescence decay curve (AUC) relative to that of a blank in which no antioxidant was present. Solutions of AAPH, fluorescein, and trolox were prepared in a phosphate buffer (75 mmol/l, pH 7.4). Samples were diluted in the phosphate buffer as well. Reaction mixture (total volume 200 *μ*l) contained FL (170 *μ*l, final concentration 5.36 × 10^−8^ mol/l), AAPH (20 *μ*l, final concentration 51.51 mmol/l), and sample, 10 *μ*l. The FL solution and sample were incubated at 37°C for 20 min directly in a microplate reader, and AAPH (dissolved in buffer at 37°C) was added. The mixture was incubated for 30 s before the initial fluorescence was measured. After that, the fluorescence readings were taken at the end of every cycle (1 min) after shaking. For the blank, 10 *μ*l of phosphate buffer was used instead of the extract. The antioxidant activity was expressed in micromole trolox equivalents (*μ*mol TE) per liter juice. Trolox solutions (6.25; 12.5; 25; and 50 *μ*mol/l) were used to define the standard curve. ORAC was carried out using a FLUOstar OPTIMA plate reader (BMG Labtech, Germany); excitation wavelength of 485 nm and emission wavelength of 520 nm were used.

### 2.7. Statistical Analysis

The data were processed by nonparametric analysis (Kruskal-Wallis test). Statistical significance between experimental groups was determined by u-criterion of Mann-Whitney and the differences were considered significant at *P* < 0.05. The intergroup comparison was made with one-way ANOVA. Data are presented as mean ± SD from the indicated number of experiments.

## 3. Results and Discussion

### 3.1. Chemical Characteristics and Antioxidant Activity of Black Chokeberry Juice

The physicochemical parameters and carbohydrate composition of chokeberry juice are presented in Table 1. The dry solid content of the juice is 18.1% and titratable acidity is 0.89%. The total carbohydrate content of* Aronia* juice was 14.84%. The main sugars presented in the juice were glucose, fructose, and the predominant sorbitol.

The polyphenol content and composition, as well as antioxidant activity, of chokeberry juice are shown in Table 2. Chokeberry juice is a very rich source of total polyphenol compounds, 4772.2 mg/l. Proanthocyanidins (PACNs) are the predominant phenolic compounds in the juice with total content of 3529.1 mg/l. Hydroxycinnamic acids represented by neochlorogenic and chlorogenic acids are the second most abundant polyphenols with cumulative content of 806.2 mg/l. Quercetin and quercetin glycosides, isoquercitrin (quercetin-3-glucoside) and rutin (quercetin-3-rutinoside), as well as the flavan-3-ol epicatechin are also present as minor components in the juice. The total amount of anthocyanins is 456.2 mg/l juice.* Aronia* anthocyanin profile is very simple consisting almost exclusively of cyanidin glycosides, namely, cyanidin-3-arabinoside, cyanidin-3-galactoside, cyanidin-3-glucoside, and cyanidin-3-xyloside. Cyanidin-3-galactoside and cyanidin-3-arabinoside are the predominant representatives with a cumulative content >90% in the berries [32]. The high content of polyphenol compounds in the juice determines its particularly high antioxidant activity measured by the ORAC method, 55307 *μ*mol TE/l. This method measures the ability of the antioxidant to scavenge peroxyl radicals via hydrogen atom transfer. These radicals are physiologically the most important ones and the hydrogen atom transfer is the most physiologically relevant mechanism of antioxidant action. One of the first attempts to quantify dietary antioxidant needs of the body demonstrated that consumption of certain berries and fruits such as blueberries, mixed grape, and kiwifruit was associated with increased ORAC plasma antioxidant capacity in the postprandial state and consumption of an energy source of macronutrients containing no antioxidants was associated with a decline in plasma antioxidant capacity [33]. It was estimated that, according to the energy intake of the diet, 5000–15000 *μ*mol TE/g is necessary to cover human daily antioxidant needs. The high ORAC result of chokeberry juice indicates that this product is a very rich source of dietary antioxidants.

### 3.2. Influence of Chokeberry Juice Intake on Somatometric Parameters and Lipid Profile of Rats

Aging is associated with undesirable changes in body composition that expose older individuals to a number of metabolic complications. It is well-known that body fat increases with age and is preferentially accumulated in the abdominal region (i.e., visceral adiposity), thereby contributing to the development of metabolic decline in aging rats and humans, cardiovascular diseases, and diabetes in older adults. As they age, rats exhibit linear growth, and both lean and fat mass increase until late middle to early old age. Fat mass consists primarily of adipose tissue, but lean mass includes organs, tendons, cartilage, blood, and body water in addition to skeletal muscle [34]. Fat mass is defined as the sum of the adipose pads (mesenteric, retroperitoneal, epididymal, abdominal, and subcutaneous).  Table 3 presents the somatometric parameters studied. Our results showed that weight and abdominal circumference of old animals were significantly higher than those of young controls, which can be attributed to the natural growth process. Group A showed a mean body weight higher than that of the CO group (*P* < 0.05). No significant differences were observed in the abdominal circumference among the old controls and the supplemented animals. The rats ingesting* Aronia* juice had a significantly higher BMI as compared to the CO group (*P* < 0.05) which is above the normal limits (0.45–0.68 g/cm^2^) [18].

Lee considered values greater than 0.31 as an indicator of obesity and in our investigation all groups studied were within this threshold [18, 35]. We did not measure the amount of adipose tissue in the body, since it was not in the scope of our study, and we could evaluate it only indirectly based on somatometric indices. The increased body weight of the animals is likely to be a result of the increased carbohydrate intake, since the juice ingested was in unlimited amounts, a total dose of 64 ml/kg. As it was already mentioned, the carbohydrate content of the juice was approximately 15%. In this first experiment, the juice dosage administered was relatively high and not applicable to humans. We have already planned further experiment with a reduced dose of* Aronia* juice applicable to humans. The higher body weight of the supplemented animals is likely to be due not only to obesity but to an increased relative portion of nonfat body mass as well. According to additional data (not presented in this paper) the weights of liver, heart, and spleen showed significantly higher values in the supplemented animals as compared to the old controls (*P* < 0.05) without histopathologic signs of obesity of these organs. We found a strong and positive correlation between the animal weight and the weights of the heart, *r* = 0.77, the liver, *r* = 0.90, and the spleen, *r* = 0.69. The changes in the somatometric parameters observed in our experiment were similar to those in other studies [18, 34, 36].

**Figure 1:**
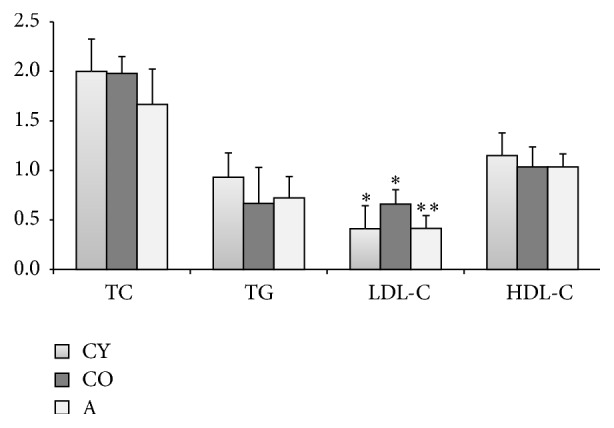
Lipid profile of chokeberry juice-supplemented animals compared to old and young controls. Different number of asterisks indicates significance at level *P* < 0.05.

Alterations in BMI were associated with dyslipidemic profile and oxidative stress in the serum of rats; therefore, BMI may predict these adverse consequences of the obesity in rats [18]. Paradoxically, treatment with* Aronia* juice not only normalized the lipid profile of the supplemented animals, but also contributed to its optimization by significantly lowering the proatherogenic low-density cholesterol (Figure 1). The figure demonstrates the parameters of the lipidogram of the animals from the three experimental groups.

Age and gender are physiologic factors that have a marked influence on plasma lipid levels in several species. Dyslipidemia is characterized by increased triglyceride and/or low-density lipoprotein levels, as well as declined high-density lipoprotein levels. Of particular interest is the fact that plasma levels of total cholesterol and LDL cholesterol are well known to increase with normal aging, while HDL cholesterol declines with age. The potential mechanisms of age-related disorders of lipoprotein metabolism in both humans and animals are related to changes in the liver sinusoidal endothelium, postprandial lipemia, insulin resistance induced by free fatty acid, growth hormone, androgen (only for men), and expression and activity of peroxisome proliferator-activated receptor [37]. Our results confirmed these findings, since in old rats there was evidence of partial dyslipidemia, expressed in increased levels of LDL-C (*P* < 0.05), as well as a tendency of decreased HDL-C, as compared to young animals. The treatment of old rats with* Aronia* juice led to a 16.5% decrease in total cholesterol (TC) in comparison to the untreated animals, whereas there was no significant difference in TC of old and young controls. With regard to the LDL-C, young controls showed 30% decrease, as compared to old controls (*P* < 0.05), and supplemented animals showed a 36% reduction, as compared to adult controls (*P* < 0.05). High-density lipoprotein cholesterol values of young controls were 10% higher in comparison to elderly controls, while groups CO and A did not show any difference. These data show that intake of black chokeberry juice significantly reduced the proportion of atherogenic LDL-C fraction in blood but did not affect the values of HDL-C. Influencing the age-related dyslipidemia with chokeberry juice is a possibility to correct the senescence-related metabolic changes via nonpharmacological approach, thus making a step towards healthy aging. In hyperlipidemias, either spontaneous or induced, the effects of black chokeberry juice are more demonstrative. According to Valcheva-Kuzmanova et al.,* Aronia melanocarpa* fruit juice significantly hindered the dietary-induced elevation of plasma total cholesterol, LDL-C, and triglycerides in hyperlipidemic rats and this effect was attributed to the high content of phenolic phytochemicals in the juice [13–15]. Similarly to our findings, neither the high-cholesterol feeding nor the administration of* Aronia melanocarpa* fruit juice caused significant changes in the concentrations of plasma HDL-C in these studies. Probable mechanisms involved in the lipid lowering effects of flavonoids might include inhibition of cholesterol absorption as demonstrated for silymarin and tea catechins, improved catabolism of lipoproteins rich in triglycerides as demonstrated for cyanidin, and increase in the bile flow, biliary bile cholesterol, and biliary bile acids as demonstrated for naringin [13]. Other mechanisms are inhibition of the enzyme 3-hydroxy-3-methylglutaryl-CoA reductase leading to a reduction in the cholesterol synthesis, as well as to inhibition of the enzyme acyl-CoA:cholesterol acyltransferase leading to a decrease in the cholesterol esterification in the intestine and liver, and a subsequent decrease in its absorption and inclusion into lipoproteins. Such enzyme-inhibiting activities have been demonstrated for different flavonoids including quercetin, which is present in chokeberry juice [15].

### 3.3. Influence of Chokeberry Juice Intake on the Atherogenic and Cardioprotective Indices

Individual serum lipid fractions give us information about the probability of developing atherosclerosis and cardiovascular disease. However, some clinical indices calculated as the ratio between pro- and antiatherogenic lipid fractions in blood plasma have a much higher predictive value. Increased serum concentrations of LDL cholesterol and triglycerides are atherogenic and have been recognized as a risk factor for cardiovascular diseases. The increased HDL-C has been considered cardioprotective. Recent studies have shown that HDL-C promotes the reverse cholesterol transport in which HDL-C induces efflux of excess accumulated cellular cholesterol and prevents the generation of an oxidatively modified LDL-C [8]. Therefore, many authors assume the calculation of these indices as more objective when assessing the atherogenic risk and the corresponding protective effect of natural compounds on test animals [8, 20–22]. This approach has been supported by several epidemiologic studies demonstrating that TC-C/HDL-C and LDL-C/HDL-C ratios are better predictors of atherosclerosis and cardiovascular disease than any other single lipid marker. The better ability of lipid ratios to predict cardiovascular disease compared to any single lipid marker is of particular clinical relevance and can possibly be explained by the association of lipid ratios with a cluster of cardiovascular risk factors that are at least in part unrelated to cholesterol metabolism [19]. In spite of controlling LDL-C with statins, patients may still have residual dyslipidemia, which can be partially attributed to triglyceride-rich lipoproteins, especially remnant lipoproteins [38]. Therefore, assessing atherogenic dyslipidemia involves an index showing the levels of triglyceride-containing fractions (non-HDL-C and remnant C). Triglyceride-rich lipoproteins containing a single molecule of apolipoprotein B are released into the circulation by the liver or intestine and bind the lipoprotein lipase to the surface of endothelial cells. Lipolysis of triglyceride-rich lipoproteins by lipoprotein lipase yields a triglyceride-depleted but cholesterol-rich remnant lipoproteins. Remnant lipoproteins (RLPs) contain 5 to 20 times more cholesterol per particle than LDL-C and are able to cross the endothelial barrier. Importantly, unlike native LDL-C, RLPs can be taken up in an unregulated fashion by scavenger receptors expressed by resident macrophages in the subendothelial space, facilitating foam cell formation and atherosclerosis [39]. Non-high-density lipoprotein cholesterol (non-HDL-C) is the difference between the total cholesterol concentration and the HDL cholesterol concentration, providing an estimate of cholesterol in the atherogenic particles including IDL-C, VLDL-C, Lp(a)-C, and LDL-C [39, 40].

**Figure 2:**
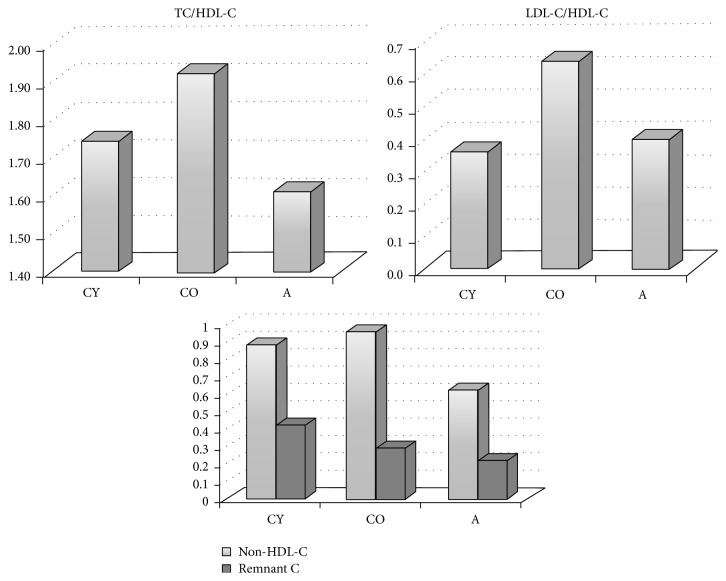
Distribution of mean values of atherogenic indices in the groups studied.

Figure 2 depicts the atherogenic indices for all studied groups. To our knowledge, such atherogenic indices are calculated for the first time after supplementation of* Aronia* products. All indices calculated for the group of young animals are related to lower risk as compared to the old animals, which is a natural expression of the aging process, results confirming the adequacy of the experimental model chosen. Similar reduced risk was observed in the group of* Aronia*-supplemented animals, demonstrated by all the indices.

**Figure 3:**
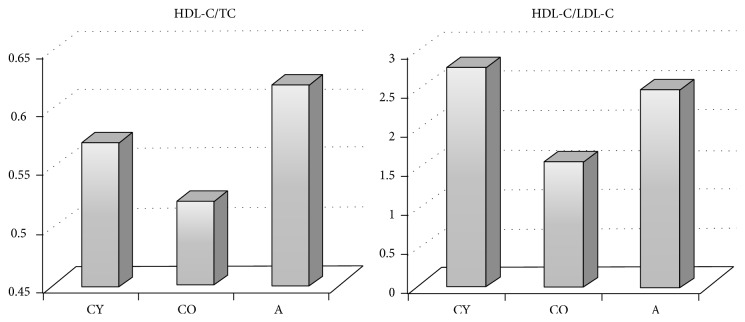
Distribution of mean values of cardioprotective indices in tested groups.

HDL-C is the only atheroprotective fraction of the routine lipid profile, but there is evidence that its elevation (70% to 100%) is not beneficial and even can increase the cardiovascular risk. Therefore, nonpharmacological measures to influence HDL-C values are particularly relevant. Cardioprotective indices calculated as ratios of HDL-C are a basis for a better prognosis. Therefore, apart from the atherogenic indices, we calculated cardioprotective indexes as well expressed as ratios between HDL-C and TC or HDL-C and LDL-C (Figure 3). Our results indicate that the supplementation of* Aronia* juice markedly improves the cardioprotective indices of the animals studied. Moreover, the cardioprotective indices of these animals are very close to those in young controls. Thus, it can be concluded that atherogenic indices in* Aronia*-supplemented animals clearly show lower atherogenic risk whereas cardioprotective indices show optimization of the lipid profile, which indicate protection of cardiovascular system.

### 3.4. Effect of Chokeberry Juice Intake on the Age-Related Changes of Aorta

Dobiášová et al. (2011) have found that the atherogenic index is strongly correlated with the size and concentration of individual lipoprotein subpopulations, as well as the vascular changes, and has a high predicting potential in determining cardiovascular risk [41].

**Figure 4:**
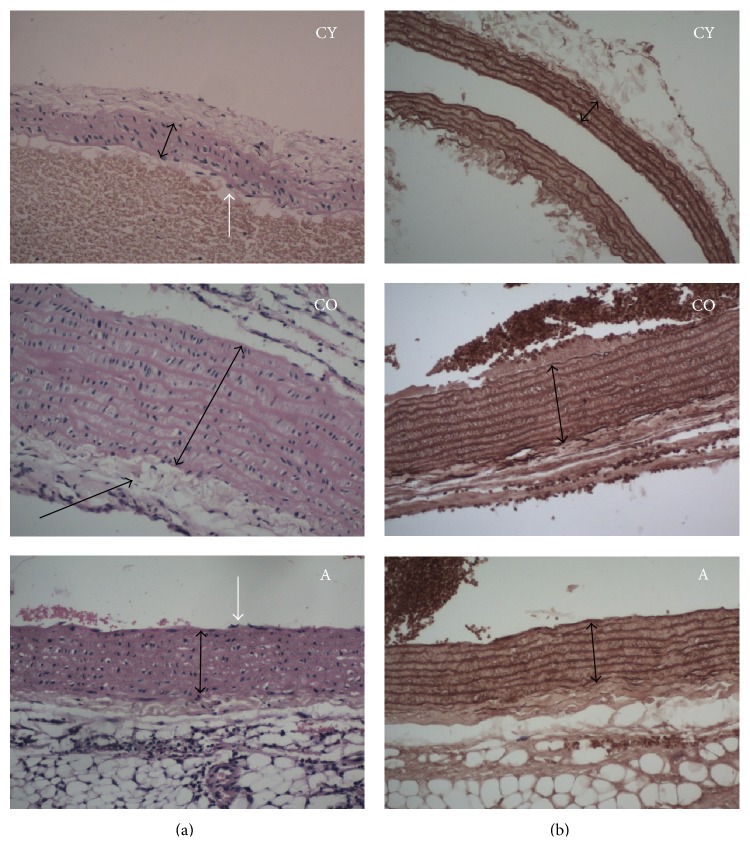
Aortic walls stained with hematoxylin/eosin (×200) (a) and orcein (×200) (b). Black arrow on the CO image indicates* tunica intima* thickening, bidirectional arrows indicate* tunica media,* and white arrow indicates normal* tunica intima*.

Age-related structural changes in the cellular and extracellular components of the blood vessels have a bearing on their function. According to data of Wheeler et al. (2015) aging has been associated with fibrosis in a number of organs including the aorta. An increase in collagen content has been reported to occur in aortas from older subjects [42]. Although elastin content has been reported to be similar in aortas from young and older human subjects, the abundance of elastin relative to other extracellular matrix components is reduced in the aortas from old subjects. The same study demonstrated that the diameter, luminal perimeter, and wall thickness of the thoracic aorta were increased as a function of age. In another study, Greenwald (2007) reported that conduit arteries become stiffer with age because elastin becomes fragmented, degraded, and replaced by much stiffer collagen. Furthermore, both proteins become stiffer because of cross-linking and calcification, and these changes are accelerated by uraemia, hyperglycaemia, and oxidative stress [43]. As age increases, the wall thickness of the aorta increases, which is mainly due to the thickening of the middle layer that in turn thickens because of the increase in collagen content in the aortic wall. In our study, supplementation of* Aronia* juice resulted in retardation of age-related changes in the aortic wall.  Figure 4 demonstrates the changes in the aortic wall observed in the three groups of animals.

From Figure 4, it is evident that aortic wall thickness is greater in old versus young rats, which can be attributed to the natural aging process. In young controls,* tunica intima* is intact, whereas in old controls a focal subendothelial deposition of lipid droplets was observed (black arrow), as well as lack of the endothelial layer at some sites.* Tunica media* in young controls is represented by 4-5 smooth, thick, elastic membranes, intensely dyed by orcein. The* tunica media* in old controls in haematoxylin-eosin staining showed focal hyperplasia and hypertrophy of the smooth muscle cells, mainly expressed in the inner third. The orcein staining in old controls showed loosening and fragmentation of the paler-colored elastic membranes, a finding not present in young controls and supplemented animals. In* Aronia*-treated animals, intact endothelium and absence of subendothelial lipid droplets were observed in* tunica intima*. The* tunica media* of those animals revealed hypertrophy of single smooth muscle cells. Orcein staining in the same group showed smooth and straight elastic membranes with preserved integrity and staining intensity closer to that of young controls.

## 4. Conclusion

The importance of the present study is associated with a potential application of its findings in human nutrition and preventive medicine. Its results indicate that black chokeberry (*Aronia melanocarpa*) juice improves lipid profile of supplemented animals and slows down the age-related changes in the aortic wall. The analysis of the atherogenic and cardioprotective indices definitely confirms that chokeberry juice has antiatherogenic and cardioprotective effects and can be recommended as a prophylactic means for healthy aging.

## Figures and Tables

**Table 1:** Physicochemical parameters and carbohydrate composition of black chokeberry juice.

Dry solids, %	Titratable acidity, %	pH	Carbohydrates		
			Glucose, %	Fructose, %	Sorbitol, %
18.1	0.89	3.5	3.69	2.15	9.00

**Table 2:** Polyphenol content and composition and antioxidant activity of chokeberry juice.

Neochlorogenic acid, mg/L	Chlorogenic acid, mg/L	Epicatechin, mg/L	Rutin, mg/L	Isoquercitrin, mg/L	Quercetin, mg/L	ACN content, mg/L	PACN content, mg/L	Polyphenol content, mg/L	ORAC, *µ*mol TE/L
415.7	390.5	40.2	70.9	21.4	64.4	456.2	3529.1	4772.2	55307

**Table 3:** Somatometric indices.

Parameters	Groups		
	CY	CO	A
Weight (g)	104.29 ± 6.5%^a^	363.60 ± 6.99%^b^	419.17 ± 42.63%^c^
Abdominal circumference (cm)	12.64 ± 1.25%^a^	18.67 ± 0.98%^b^	19.33 ± 1.54%^b^
BMI (g/cm^2^)	0.41 ± 0.04%^a^	0.61 ± 0.02%^b^	0.73 ± 0.04%^c^
Lee index	0.30 ± 0.01%^a^	0.29 ± 0.004%^a^	0.31 ± 0.01%^a^

Results are presented as mean values ± standard deviations. The intergroup comparison is made with one-way ANOVA. Significantly different values in each row are marked with different superscript letters.
